# Successful Endoscopic Management of Nasal Septum Squamous Cell Carcinoma Following Induction Chemotherapy With Paclitaxel, Carboplatin, and Cetuximab: A Case Report

**DOI:** 10.1002/ccr3.70841

**Published:** 2025-09-15

**Authors:** Teru Ebihara, Naohiro Takeshita, Kazuhiro Omura, Nei Fukasawa, Masato Nagaoka, Nobuyoshi Otori

**Affiliations:** ^1^ Department of Otorhinolaryngology The Jikei University School of Medicine Tokyo Japan; ^2^ Department of Otorhinolaryngology Dokkyo Medical University Saitama Medical Center Tokyo Japan; ^3^ Department of Pathology The Jikei University School of Medicine Tokyo Japan

**Keywords:** carboplatin, cetuximab, induction chemotherapy, paclitaxel, sinonasal cancer

## Abstract

This case provides evidence of the efficacy of induction chemotherapy with paclitaxel, carboplatin, and cetuximab in sinonasal squamous cell carcinoma. Treatment‐induced tumor shrinkage can expand the working space in the complex and narrow sinonasal region, potentially avoiding the need for extensive surgeries that require reconstruction.

## Graphical abstract

En bloc resected nasal septal squamous cell carcinoma after induction chemotherapy. Coronal slabs demonstrate marked tumor shrinkage along the cranial–caudal axis. Red asterisks: microinvasive carcinoma in situ. Blue lines: inflammation/foreign‐body reaction where the tumor disappeared owing to chemotherapy. Yellow asterisks: dysplastic epithelium requiring distinction from regenerative change. Green arrows: septal perforation and middle turbinate. All margins negative.

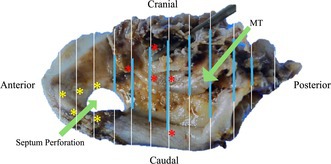

## 1 Introduction

Induction chemotherapy for sinonasal cancer lacks consensus owing to its rarity and the exclusion of such cases from most phase III studies on head and neck squamous cell carcinoma (HNSCC). Common treatment regimens include docetaxel/cisplatin/5‐fluorouracil (TPF), cisplatin, carboplatin/etoposide, and cyclophosphamide/doxorubicin/vincristine. A 2011 retrospective review by Hanna et al. revealed that 67% of 46 patients with advanced sinonasal squamous cell carcinoma responded to induction chemotherapy, with 9% exhibiting stable disease [1]. Another review in 2014 of 41 patients with unresectable maxillary sinus squamous cell carcinoma showed 2‐ and 3‐year overall survival rates of 41% and 35%, respectively, after induction chemotherapy followed by various treatments, indicating a clinical benefit with manageable toxicity [2]. Induction chemotherapy reportedly preserves the orbit in patients with sinonasal cancer with orbital invasion [3]. However, a meta‐analysis found that patients who received TPF induction chemotherapy before cisplatin‐based chemoradiotherapy (CRT) were at a higher risk of myelosuppression and incomplete CRT [4, 5].

A Japanese multicenter phase II trial examined the efficacy of induction chemotherapy with paclitaxel, carboplatin, and cetuximab (IC‐PCE) in patients with unresectable locally advanced HNSCC (LA‐HNSCC). The trial reported an 88.6% response rate with no effect on compliance with subsequent CRT, suggesting IC‐PCE's feasibility and effectiveness [6]. Although the results of treating head and neck cancers appear promising, data on IC‐PCE's efficacy in sinonasal cancers remain unreported.

Herein, we report a case of unresectable locally advanced nasal septal squamous cell carcinoma that was successfully downsized by IC‐PCE and completely resected via an endoscopic approach. We provide a detailed pathological evaluation of the tumor reduction process following IC‐PCE.

## 2 Case History/Examination

**FIGURE 1:**
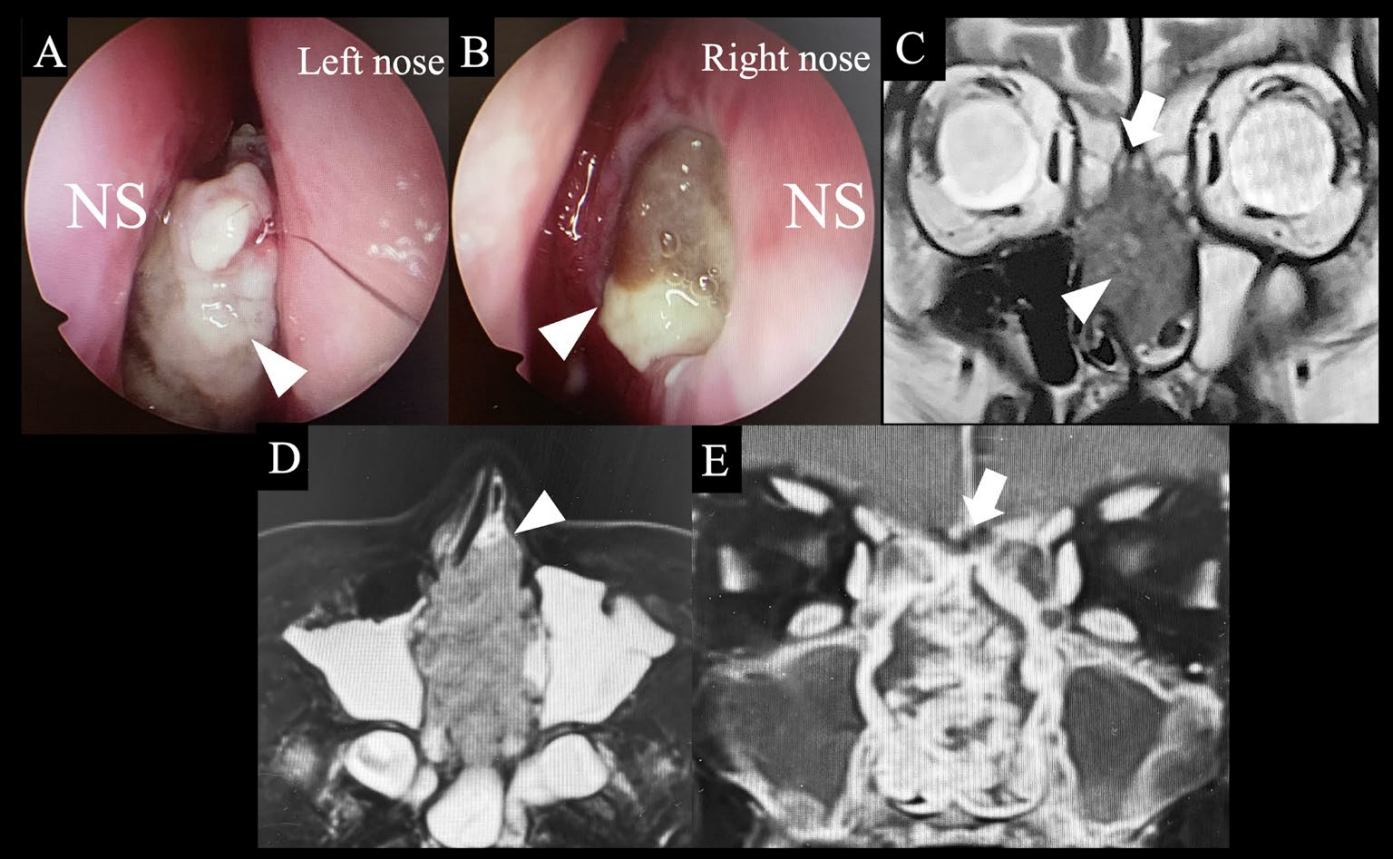
Endoscopic endonasal images before induction chemotherapy. Left (A) and right (B) images. Tumor occupying the nasal cavity, predominantly on the left side is observed (arrowhead). The tumor on the left side is also visible on the right side through perforation of the nasal septum. Coronal (C), axial (D) T2‐weighted and coronal contrast‐enhanced TSE DIXON (E) magnetic resonance image obtained before induction chemotherapy. The tumor is centered on the nasal septum (C arrowhead) and extended anteriorly to the margin of the piriform aperture (proximally only, no mucosal or skin invasion, D arrowhead), cephalad to the cribriform plate (proximally only, no obvious invasion, C and E arrow), lateral to the bilateral middle turbinate and posterior to the front of the anterior wall of sphenoid sinus. NS: Nasal septum.

**FIGURE 2:**
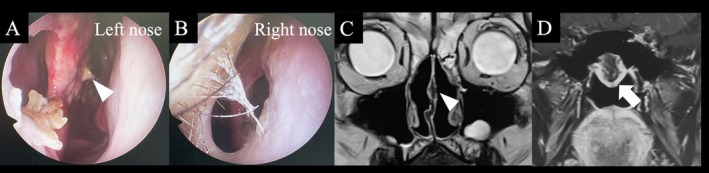
Endoscopic endonasal images after induction chemotherapy. Left (A) and right (B). Significant tumor reduction is observed. Residual tumor is noted in the posterior nasal septum (arrowhead). (C) (D) Coronal T2‐weighted magnetic resonance image obtained after induction chemotherapy. Significant tumor shrinkage is observed. A partially thickened area posterior to the nasal septum is suspected to have residual tumor (arrowhead), and there is clear residual tumor at the posterior end of the nasal septum (arrow).

**FIGURE 3:**
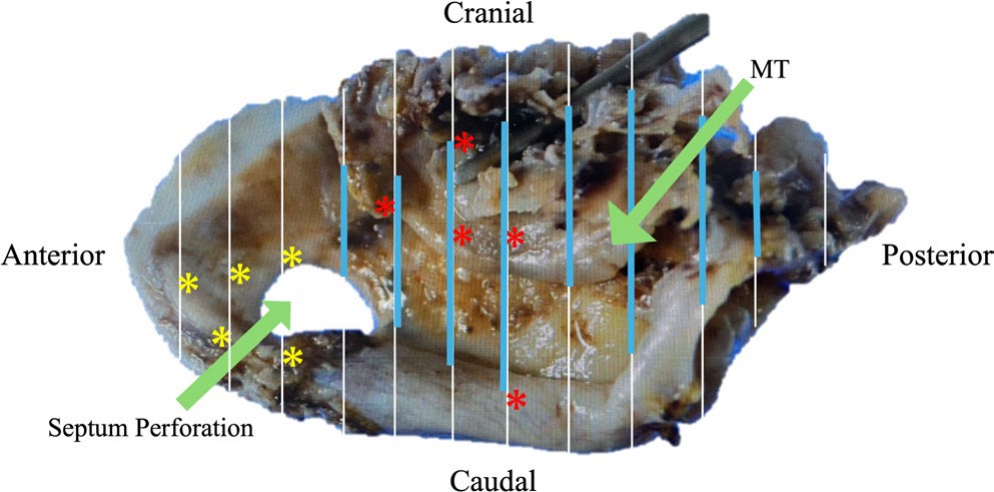
En bloc resected tumor specimen. Specimen viewed from the left side. Red asterisk: Area with microinvasive carcinoma in situ. Blue line: Area with inflammation and foreign body reaction where the tumor disappeared owning to chemotherapy. Yellow asterisk: Area with dysplastic epithelium requiring differentiation from regenerative changes. All resection margins are negative. MT: Middle turbinate.

**FIGURE 4:**
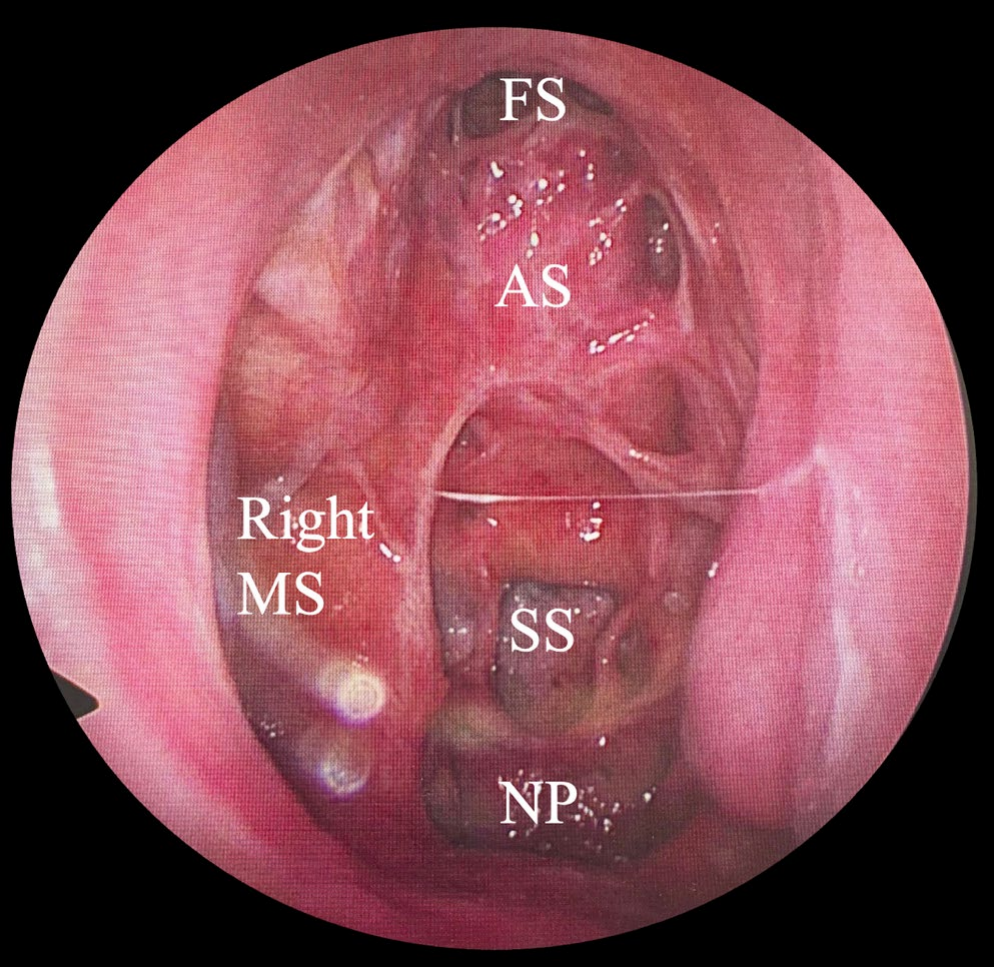
Zero‐degree endoscopic endonasal image 9 months after operation from left nasal cavity. No crust retention was observed. AS: Anterior skull base, FS: Frontal sinus, MS: Maxillary sinus, NP: Nasopharynx, SS: Sphenoid sinus.

A 59‐year‐old woman with nasal septal perforation presented with nasal obstruction and postnasal drip. ECOG performance status was 0 and she had no obvious organ dysfunction. A tumor was identified in the left nasal cavity, and a biopsy confirmed squamous cell carcinoma (G2, moderately differentiated) [7]. The tumor was centered on the nasal septum and extended anteriorly to the margin of the piriform aperture (proximally only, no mucosal or skin invasion), cephalad to the cribriform plate (proximally only, no obvious invasion), lateral to the bilateral middle turbinate, and posterior to the front of the anterior wall of the sphenoid sinus (cT3N0M0). (Figure 1). Resection by endoscopic approach alone was not chosen due to the possibility of cutting into the tumor due to working space issues. IC‐PCE was initiated followed by surgery. The regimen consisted of carboplatin at an area under the curve of 1.5, paclitaxel at 80 mg/m^2^, and cetuximab at an initial dose of 400 mg/m^2^, followed by 250 mg/m^2^ weekly for 8 weeks [6]. Considering the article by Enokida et al. [6], 8 courses were scheduled, and all courses were completed without delay, and only grade 2 leukopenia and nausea were observed. After eight courses of PCE, the percentage of tumor shrinkage was 41%, which was a partial response [8]. After significant tumor shrinkage (Figure 2), surgery was performed under general anesthesia using an endoscopic approach. The anterior end of the nasal septum cartilage was resected as the anterior margin and the cribriform plate as the cephalic margin. The bilateral margin was resected from the lateral wall of the nasal cavity. The nasal floor mucosa was excised as the caudal margin and the nasopharyngeal tissue as the posterior margin. We could resect the tumor en bloc (Figure 3). Intraoperative and permanent pathologic diagnoses were margin negative. Nine months have passed since the surgery; we observed no nasal blockage or crusts and good epithelialization inside the nose (Figure 4). CT scans are performed every 3 months to follow up with the patient, with no signs of recurrence or metastasis to date.

## 3 Discussion

Several studies have reported the efficacy of surgery after induction chemotherapy for HNSCC. Yokota et al. [9] performed minimally invasive surgery in 90.2% of patients with HPV‐positive oral squamous cell carcinoma after induction chemotherapy, avoiding the need for free‐flap reconstruction and tracheostomy. A pathological complete response was achieved in 73.2% of cases, and adjuvant radiation therapy was unnecessary in 85.0% of cases [9]. Another study by Arai et al. reported that IC‐PCE in tongue squamous cell carcinoma avoided reconstruction surgery in seven out of eight cases, achieving complete resection with negative margins [10]. A report indicated that all patients with sinonasal squamous cell carcinoma who received preoperative chemotherapy after induction chemotherapy with CDDP‐5‐FU had negative surgical margins, demonstrating the efficacy of surgical margins [11]. However, no comprehensive reports on the efficacy of IC‐PCE for sinonasal cancer are available. To the best of our knowledge, this case report is the first to demonstrate the efficacy of surgical resection after IC‐PCE for sinonasal cancer.

Traditional induction chemotherapy for sinonasal cancer is associated with strong myelosuppression, which poses the risk of incomplete CRT. One common induction chemotherapy regimen is the TPF regimen, which is not only well established with evidence but has also the disadvantage of frequent adverse events such as myelosuppression [12]. In contrast, Takeshita et al. reported a lower incidence of grade 3/4 adverse events during IC‐PCE treatment for nasopharyngeal carcinoma, which suggests improved compliance with subsequent CRT [13]. Because of the possibility that the next treatment, surgery, could not be completed due to adverse events, we chose the PCE regimen, which is based on evidence from phase II trials but is safer and has a similarly high response rate. In this case, for the first time, we administered IC‐PCE to sinonasal squamous cell carcinoma and observed no grade 3 or higher adverse events, which allowed for safe surgery. The feasible toxicity profile of IC‐PCE is useful for advancing treatment without delay.

Endoscopic approaches are widely accepted for sinonasal cancer treatment owing to advancements in techniques over the past 30 years. Although operating on tumors with wide margins in a confined nasal cavity is unrealistic, a review of 7808 patients with sinonasal squamous cell carcinoma showed improved overall survival with surgical resection and negative or microscopically positive margins when compared with nonsurgical treatments. However, no improvement was observed in cases with macroscopically positive margins [14]. In the surgical treatment of sinonasal cancer, the challenge lies in securing adequate margins for resection without compromising the confined working space of the nasal cavity.

In this case, significant tumor shrinkage following IC‐PCE allowed for detailed margin evaluation, particularly in the posterior nasal cavity areas that are often blind in open approaches. Compared with external incisions, the endoscopic approach has the advantage of allowing a more detailed margin evaluation in the posterior nasal cavity and near the skull bases. Endoscopic surgery after IC‐PCE may be a good treatment strategy to exploit these benefits.

**FIGURE 5:**
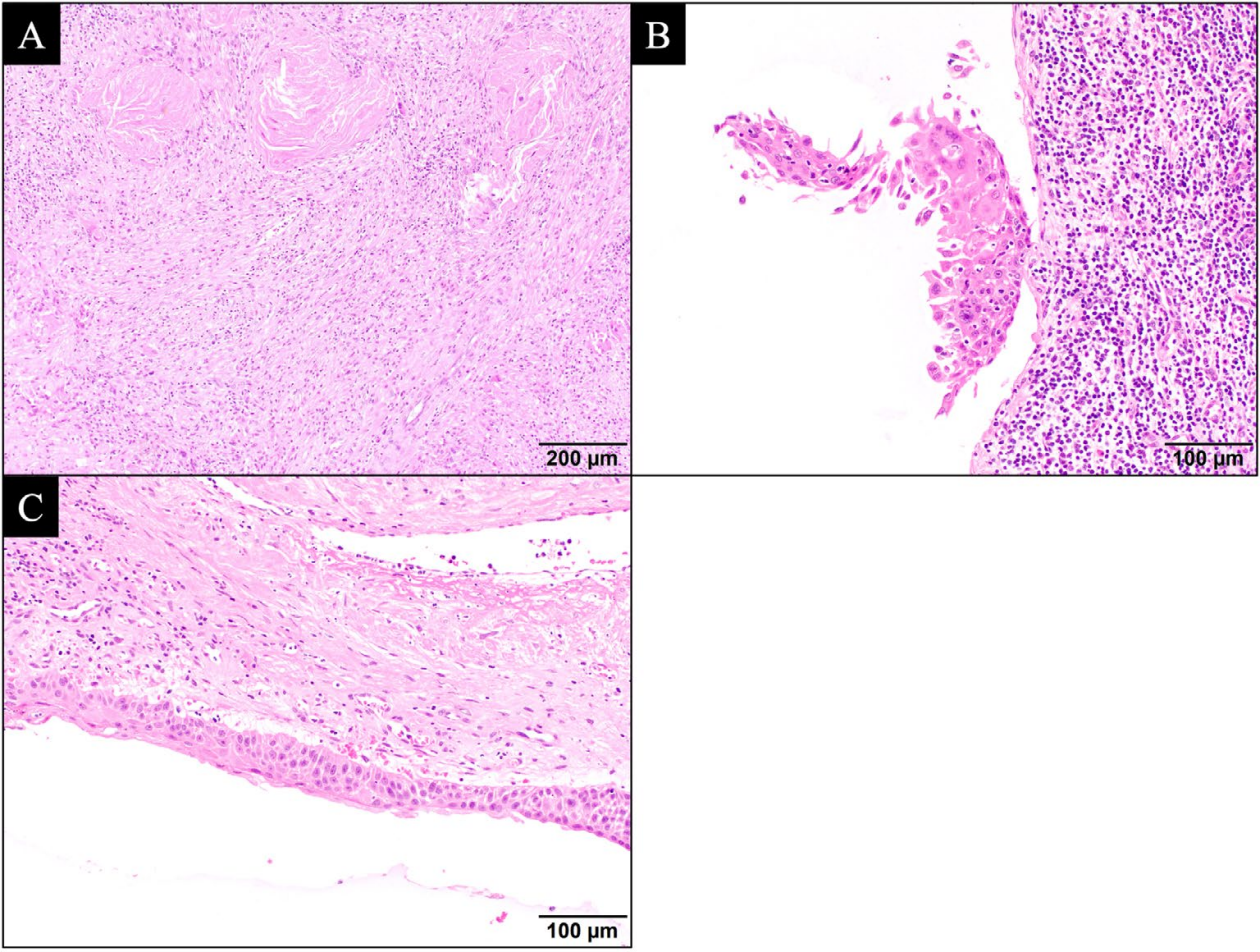
Histopathological image of en bloc resected tumor specimen. (A) Histopathological image (hematoxylin staining) of the area with inflammation and foreign body reaction involving macrophages, where the tumor had disappeared owning to chemotherapy. (B) Histopathological image (hematoxylin staining) of the area with carcinoma in situ. (C) Histopathological image (hematoxylin staining) of the area with dysplastic epithelium requiring differentiation from regenerative changes.

The en bloc resected specimen (Figure 3) enabled the visualization of the tumor reduction process, in turn demonstrating IC‐PCE's effectiveness. The mapping results showed no invasive carcinoma. Most areas showed inflammation and foreign body reaction owing to chemotherapy (Figure 5A). A majority of the carcinoma in situ (Figure 5B) was observed in areas where the tumor was macroscopically present, with some observed at the tumor periphery where the tumor had disappeared macroscopically. We observed dysplastic epithelium (Figure 5C) (which required differentiation from the tumor) in areas where the tumor had disappeared macroscopically. Because predicting the residual form of the tumor is difficult before chemotherapy, selecting a surgical approach that ensures complete resection in areas where the tumor was present before chemotherapy is necessary. Good surgical candidates following IC‐PCE include individuals wherein the tumor spread allows for surgical resection, but with limited working space. Patients requiring external incisions or reconstructive surgery with potential cosmetic and functional impairments should also be considered.

The limitation of this case report is the short follow‐up period of 9 months after surgery. However, the strength of this case report is that it entails the first evidence of the efficacy of IC‐PCE in sinonasal cancer and suggests that tumor shrinkage induced by IC‐PCE can expand the working space in the complex and narrow sinonasal region. This treatment regimen may help potentially avoid the need for extensive surgeries that require reconstruction.

## Author Contributions

**Teru Ebihara:** conceptualization, data curation, formal analysis, investigation, project administration, visualization, writing – original draft, writing – review and editing. **Naohiro Takeshita:** conceptualization, project administration, writing – review and editing. **Kazuhiro Omura:** conceptualization, data curation, formal analysis, writing – review and editing. **Nei Fukasawa:** data curation, formal analysis, writing – review and editing. **Masato Nagaoka:** conceptualization, formal analysis, methodology, writing – review and editing. **Nobuyoshi Otori:** project administration, writing – review and editing.

## Consent

Written informed consent was obtained from the patient to publish this report in accordance with the journal's patient consent policy.

## Conflicts of Interest

The authors declare no conflicts of interest.

## Data Availability

The data that support the findings of this study are available from the corresponding author upon reasonable request.
